# Evaluation of the impact of a socio-educational intervention in quality of life and mental health of institutionalized elderly

**DOI:** 10.1192/j.eurpsy.2021.1243

**Published:** 2021-08-13

**Authors:** A.P. Amaral, A. Loureiro

**Affiliations:** Coimbra Health School, Polytechnic of Coimbra, Coimbra, Portugal

**Keywords:** mental health promotion, PSYCHOLOGICAL WELL-BEING, quality of life, Elderly

## Abstract

**Introduction:**

With the huge increase of life expectancy in developed countries, new needs for long-term care arise in order to guarantee an active ageing for an increasing older adult population. One way to promote emotional well-being and quality of life in elderly is through socio-educational interventions.

**Objectives:**

To test the effects of a socio-educational intervention in quality of life and mental health of institutionalized elderly.

**Methods:**

This study employed a pretest-posttest design. Measures: Portuguese version of Mental Health Inventory (Ribeiro, 2001) and WHOQOL-OLD (Vilar, Sousa & Simões, 2009). Qualitative assessment was made using a logbook. Participants: 15 institutionalized elderly, 60% females, with mean age of 82.5 years (sd=8.5). The intervention ran for 2 months, with 12 group sessions, 60 minutes each, held twice a week. A nonparametric paired samples tests was conducted to evaluate the impact of the intervention.

**Results:**

After the intervention, results showed a significant increase of total value of mental health (p=.021). Concerning dimensions: significant increase of positive psychological well-being (p=.014), emotional ties (p=.050), positive affect (p=.004), behavioural emotional control (p=.018), and a significant decrease of depression (p=.043). Concerning quality of live, the results showed a significant increase of the mean values of the facets: social participation and intimacy (p=.005; p=.027, respectively).

**Conclusions:**

Overall, the intervention implemented with institutionalized elderly had good results, with significant increase of positive psychological well-being and decrease of depression. Although there was no control group, the results suggest that the socio-educational intervention implemented can contribute to promote mental health in elderly.

